# Imaging of Renal Medullary Carcinoma

**DOI:** 10.15586/jkcvhl.2017.62

**Published:** 2017-03-21

**Authors:** Federico Greco, Eliodoro Faiella, Domiziana Santucci, Carlo Augusto Mallio, Marco Nezzo, Carlo Cosimo Quattrocchi, Bruno Beomonte Zobel, Rosario Francesco Grasso

**Affiliations:** Unit of Diagnostic Imaging, Università Campus Bio-Medico di Roma, Rome, Italy

**Keywords:** Computed tomography, contrast-enhanced ultrasound, magnetic resonance imaging, renal cell carcinoma, renal medullary carcinoma

## Abstract

Renal medullary carcinoma (RMC) is a rare, highly aggressive tumor recognized as an independent pathological entity. African-descent adolescents and young adults with sickle cell hemoglobinopathy are the most affected groups. This rare subtype of renal cell carcinoma has its own morphogenetic and pathological characteristics. The major clinical manifestations include gross hematuria, abdominal or flank pain, and weight loss. The prognosis is very poor, with 95% of cases diagnosed at an advanced stage of the disease. In this review, we summarize the morphologic and dynamic characteristics of RMC under various imaging modalities such as ultrasound, computed tomography, and magnetic resonance. Differential diagnosis and management strategies are also discussed.

## Introduction

Renal medullary carcinoma (RMC) is a rare, highly aggressive tumor considered an independent pathological entity (1, 2). Although African-descent adolescents and young adults with sickle cell (SC) hemoglobinopathies are the most affected, there are reports of RMC in non–African-American patients without hemoglobinopathy (1, 3). Men are affected more often than women (M:F = 2:1) (1, 3). Some authors consider RMC to be a subtype of collecting duct carcinoma (4). Both tumors are derived from the renal medulla, biologically aggressive, and characterized by an infiltrative growth pattern (1, 2). Regarding tumor genesis, a relationship between hypoxia and angiogenesis has been suggested (4). Hypoxia increases the expression of hypoxia-inducible factor (HIF). It determines tumor protein p53 production, which induces apoptosis. In tumors lacking p53, however, HIF induces vascular endothelial growth factor leading to angiogenesis, which is necessary for the progression of disease (5). This hypothesis would explain the relationship between RMC and SC trait (4). Also, an increase in the ABL gene amplification and inactivation of the tumor suppressor gene SMARCB1 have been reported (6, 7).

This rare subtype of renal cell carcinoma (RCC) has its own morphogenetic and pathological features (8). The histologic features include reticular or yolk sac growth pattern, varying degrees of mucin production, stromal desmoplasia, and inflammatory infiltrates with lymphocytes at the margins (4). Most RMCs have hemorrhagic and necrotic areas (1). The cells have large and clear vesicular nuclei, prominent nucleoli, and dark cytoplasm with variable amounts of eosinophilic cytoplasm (9). There is frequently lymphatic and/or vascular invasion (1). Collecting duct carcinoma is characterized by cystic or papillary macroscopic appearance and tubular or papillary growth pattern. Furthermore, collecting duct carcinoma is often found in adults and is not associated with hemoglobinopathies (10). In 70% of cases, RMC is localized in the right kidney (11). The clinical presentation is characterized by gross hematuria, abdominal or flank pain, and loss of weight. The sites of metastases are locoregional lymph nodes, adrenal glands, liver, lungs, inferior vena cava, and the peritoneum (1, 12). The prognosis of RMC is very poor, and 95% of tumors are detected at an advanced stage. They are resistant to chemotherapy and biological therapy (13, 14). Simpson et al. (15) reported an average survival of 19 weeks from the time of initial diagnosis of RMC. Radical nephrectomy in patients with carcinoma *in situ* seems to prolong the survival time (16). An early and accurate diagnosis is very important, as it may improve the prognosis of patients.

Herein, we describe the morphological and dynamic characteristics of RMC under various diagnostic imaging techniques. A PubMed search was performed for the following terms: “Renal medullary carcinoma,” “Renal medullary carcinoma ultrasound,” “Renal medullary carcinoma computed tomography,” and “Renal medullary carcinoma magnetic resonance imaging.” As RMC was considered a disease entity in 1995, the search was performed for literature published between 1995 and 2016.

## Ultrasound Imaging of RMC

Accidental detection of renal lesions during abdominal ultrasound imaging is very common. About 35% of patients, in their seventh decade of life, have some form of a renal cyst (17). More than 50% of RCC are diagnosed by imaging, well before the appearance of clinical signs or symptoms (18, 19). Ultrasonographic features of RMC have been described by various authors. Khan et al. (11) described the sonographic characteristics of RMC in a 29-year-old man of Afro-Caribbean descent. The RMC was a 13-cm solid mass in the left renal pelvis with focal hydronephrosis of the upper and lower poles of the kidney. No evidence of lymphadenopathy was found at the ultrasound. Doppler study showed no flow in the renal mass. The application of power Doppler revealed minor peritumoral vascularity (11). Sathyamoorthy et al. (20) described an enlarged and diffusely echogenic right kidney, with an infiltrative mass located at the lower pole and vast lymphadenopathy, which was diagnosed as RMC. However, in other two studies, ultrasound failed to detect renal masses, and in one of these, the increased echogenicity of the medullary areas of an enlarged right kidney was misinterpreted as papillary necrosis. Two days after the ultrasound examination, the tumor was detected on computed tomography (CT) (21, 22). In another case, the ultrasound examination was negative. Two months later, the patient was diagnosed with cancer with the help of CT (23).

In the past decade, contrast-enhanced ultrasound (CEUS) made progress, allowing the study of vascularization of the renal lesions even in patients with renal insufficiency. The European Federation of Societies for Ultrasound in Medicine and Biology produced the protocols and guidelines for the use of CEUS to standardize this practice (24). Several studies have described the characteristics of renal lesions using CEUS; however, none of these studies has detected RMC. Tamai et al. (25), on histopathological examination of resected lesions conducted on 29 patients, diagnosed 26 malignant lesions (18 clear cell carcinoma, 6 papillary RCCs, 1 collecting duct carcinoma, and 1 infiltrative urothelial carcinoma) and 3 benign lesions (2 oncocytoma and 1 angiomyolipoma). Ignee et al. (26) analyzed 137 renal lesions by CEUS. No RMC was detected. Gerst et al. (27), among 34 patients studied with CEUS, diagnosed 23 clear cell carcinomas, 3 type 1 papillary carcinomas, 1 chromophobe carcinoma, 1 clear multilocular rare low-grade malignant tumor, 2 unclassified lesions, 3 oncocytomas, and 1 benign angiomyolipoma. Yong et al. (28) analyzed 74 renal lesions of 63 patients, diagnosing 22 malignant lesions. Ten of these lesions were confirmed on histology (six clear cell carcinomas, three papillary carcinomas, and one lesion showed spindle and epithelioid cells, which could not exclude an angiomyolipoma). Thus, it appears that ultrasound imaging is insufficient for the diagnosis of RMC or medullary renal lesions.

## CT Imaging of RMC

Several studies have evaluated RMC features at CT examination (Table 1) (2, 11, 20, 21, 29–32). The radiological features of RMC were first reported in 1995 by Davidson et al. (2) in five black patients, aged between 10 and 28 years, with SC trait (SCT). All five patients had advanced stage disease at diagnosis. Modes of disease spread included local direct invasion (regional lymph nodes, renal vein and inferior vena cava, liver, adrenal gland, and retroperitoneal soft tissue) and distant metastases (liver, lungs, omental lymph nodes, and pulmonary hilar lymph nodes). In all patients, the lesions were located within the renal parenchyma and involved the renal pelvis and sinus. The tumors showed an infiltrating pattern of growth; the kidneys were enlarged with the reniform aspect still preserved. The tumors surrounded and obstructed the pelvis; there was also clear caliectasis. A large area of necrosis and a heterogeneous contrast enhancement was also visible. In three of the five patients, the tumor caused caliectasis without pelviectasis. In one patient, there was communication between the necrotic cavity and the collecting system (2).

**Table 1.** CT features of RMC

References	RK	LK	N	C	Calc	H	WM	PM	RL	LiM	LuM	IH	SH	TA	TV
Davidson et al. (2)	3	1	5	3	-	-	-	-	PNS	PNS	PNS	-	-	-	PNS
Khan et al. (11)	-	1	1	1	-	-	-	-	1	-	-	-	-	-	-
Blitman et al. (21)	6	-	4	6	-	-	-	6	5	3	4	4	1	-	2
Baig et al. (31)	1	-	1	-	-	-	-	-	1	-	-	-	-	-	-
Sathyamoorthy et al. (20)	1	-	1	-	-	-	-	-	1	-	-	-	-	-	-
Neville et al. (30)	1	-	-	-	-	-	-	-	1	-	1	-	-	-	-
Raman et al. (32)	3	-	3	-	-	-	1	1	2	-	-	-	-	-	1
Shi et al. (29)	3	3	6	3	1	3	2	4	2	-	-	-	-	1	-

C, caliectasis; Calc, calcifications; H, hydronephrosis; IH, intratumoral hemorrhage; LiM, liver metastasis; LK, left kidney; LuM, lung metastasis; N, necrosis; PM, poorly defined margin; PNS, present but not specified; RK, right kidney; RL, retroperitoneal lymphadenopathy; SH, subcapsular hemorrhage; TA, thrombosis of the renal artery; TV, thrombosis of the renal vein; WM, well-defined margin.

A recent study described the clinical and CT imaging features of RMC in six patients (three women and three men; mean age, 50.5 years) (29). In three cases, the tumor was localized in the right kidney, and in the other three in the left kidney. The size of the primary lesion was between 2.90 and 10.50 cm (mean diameter, 7.48 ± 3.25 cm). In addition, three patients had hydronephrosis and caliectasis, and two had retroperitoneal lymph node metastases. SC hemoglobinopathy was present in one patient, and in the remaining five, the presence of SCT was unknown before the RMC detection. In all six cases, the tumor was in the medulla and infiltrated the renal pelvis. In four cases, they extended to the renal cortex, and in only one case, they reached the perirenal tissue. The lesions were predominantly solid and heterogeneous, with necrotic or cystic components; microcalcifications were present in only one case. The margins were well defined in two cases and poorly defined in the other four cases. The fibrous capsule was not present in any patients. In the case where the tumor reached the retroperitoneal fat, the left renal artery was infiltrated and regional lymph node metastases were present. In all cases, the attenuation of RMC on unenhanced CT was equal to normal renal cortex and medulla. On dynamic contrast-enhanced CT scan, the RMC density was much lower than the density of the normal renal cortex and medulla during all three phases enhanced: arterial (cortical) phase, cortico-medullary (medullary), and excretory phase (delayed) (29). With regards to vascularization, two angiograms, describing two kinds of lesions have been reported: hypovascular and avascular, with few small ectatic vessels surrounding the tumor (2, 11).

Khan et al. (11) described the CT features of RMC in the case of a 29-year-old man of Afro-Caribbean descent with SCT. It was a solid mass in the left kidney, with hypodense area, presumably attributable to necrosis. Medially to the lesion, a portion of compressed healthy renal parenchyma was visible. The kidney showed a thickened and irregular renal capsule; the ipsilateral psoas muscle appeared thickened. There were also some enlarged lymph nodes that moved medially and right to the aorta (11). A case of a 20-year-old African-American man showed an enlargement of the right kidney and a diffuse retroperitoneal lymphadenopathy on unenhanced CT scan (30). Dynamic contrast-enhanced CT scan confirmed the retroperitoneal lymphadenopathy and showed a diffuse enlargement with attenuation and heterogeneous enhancement of the kidney. Adjacent perinephric stranding and thickening of the renal fascias suggested an infiltrative process. Chest CT showed paratracheal and mediastinal lymphadenopathy and nodular ground-glass densities in the context of the lung parenchyma, suggesting a diagnostic hypothesis of lymphoma with renal involvement (30). Another case of a 36-year-old African-American man with SC disease showed a heterogeneous infiltrative mass of the right kidney, with extensive retroperitoneal lymphadenopathy (31).

Blitman et al. (21) analyzed six patients with SCT (three men and three women; aged from 15 to 27 years). Four were black, and two were Hispanic. The tumors were located in the central portion of the right kidney. They were hypovascular with ill-defined margins and infiltrative characteristics. Caliectasis were also present. There were enlarged retroperitoneal lymphadenopathies in five patients, whereas necrosis was present in four cases. Intratumoral hemorrhage was demonstrated in four patients, whereas subcapsular hemorrhage was present in one patient. Calcifications were absent. Thrombosis of the right renal vein was present in two, and the vascular pedicle was contained without signs of thrombosis in three of the six patients. With regard to distant metastasis, the presence of hepatic lesions was described in two patients, pulmonary lesions in three patients, liver and lung metastasis in one patient, and no metastasis in one patient at the time of diagnosis. The CT appearance of lung lesions was varying: small nodule (n = 1), cannonball lesion (n = 1) or a thick pleura-based rind (n = 1). In one case, the presence of infiltration of the renal vein was noticed during surgery, which was not noticed on the CT images (21).

In 2003, Sathyamoorthy et al. (20) reported a heterogeneous, large, infiltrating mass localized at the level of the lower pole of the right kidney, and a large retroperitoneal lymphadenopathy encasing the renal arteries, renal veins, aorta, and inferior vena cava. Three more cases were reported in 2012. A 10-year-old African-American boy had CT evidence of a hypodense, heterogeneous, large mass of the right kidney, infiltrating the right renal vein with a retroperitoneal lymphadenopathy and bone metastases. The characteristics of the lesion margins, in this case, have not been described. A 13-year-old African-American boy with SCT showed CT evidence of a hypodense mass of the right kidney with ill-defined margins and retroperitoneal, mediastinal, and supraclavicular lymphadenopathies. The patient, subjected to nephrectomy, developed bone metastases despite chemotherapy. In a 17-year-old adolescent boy with a history of SCT, the CT images showed a hypodense mass of the right kidney with well-defined margins. At the time of diagnosis, the patient had no metastatic lesions. After nephrectomy, he developed metastases to the liver, bone, and brain during chemotherapy (32).

Among all 24 patients, the most frequent CT features of RMC detected at diagnosis were necrosis of the lesion (21 patients), location in the right kidney [18 patients; in the study by Davidson et al. (2), in one of the five patients, tumor location was not specified], retroperitoneal lymphadenopathy [between 14 and 18 patients; Davidson et al. (2) did not specify how many patients among the five cases studied had the lymphadenopathies], caliectasis (13 patients), and poorly defined margins (11 patients). All the cases showed a lower frequency of lung metastases (five to nine patients), location in the left kidney (six patients), intratumoral hemorrhage (four patients), liver metastases (four to eight patients), and renal vein thrombosis (four to eight patients). More rare are well-defined margins (three patients), hydronephrosis (three patients), and subcapsular hemorrhage and calcifications (one patient) (2, 11, 20, 21, 29–32) (Table 1). In the above summary, necrosis and localization were evaluated by us both from the description and the images if they were not mentioned in the report.

## Magnetic Resonance Imaging of RMC

Few studies have described the RMC features through the use of magnetic resonance imaging (MRI). Khan et al. (11) described the MRI characteristics evaluated on T1-weighted, T2-weighted, and Short tau inversion recovery [STIR] sequences, on the axial and coronal images, describing a large central necrotic lesion, which determined caliectasis without pelviectasis, localized at the upper and lower renal poles. MRI also showed paraaortic lymphadenopathy. The aorta was displaced to the right (11). Blitman et al. (21) reported MRI findings of three patients. The study was conducted with spin-echo or gradient-echo (or both) T1-weighted sequences and T2-weighted fast spin-echo sequences with and without fat suppression in the axial and coronal planes and contrast-enhanced T1-weighted or gradient-echo sequences. The results were similar to CT in delineating the margins of lesions and to evaluate the lymphadenopathy and renal pedicle. MRI allows an easier assessment of global extent of disease and seems to be superior in detecting intratumoral hemorrhage and hepatic metastases. Raman et al. (32) described MRI features of a 17-year-old male patient with a history of SCT, showing a hypointense mass on T2-weighted images with well-defined margins and a small heterogeneous internal area on contrast-enhanced T1-weighted images on the coronal plane.

## Differential Diagnosis

In addition to collecting duct carcinoma, there are other neoplastic and non-neoplastic diseases that can mimic the central infiltrative RMC activity. Renal lymphoma can show focally or diffusely infiltrative pattern of growth (33). In children, the location is often multifocal and bilateral (34). Renal involvement occurs also in non-Hodgkin’s lymphoma, and it is visible only in 5% of patients at initial staging (33). Another aggressive tumor with infiltrative pattern is rhabdoid tumor of the kidney, which comes with a peritumoral collection in 75% of cases (35). Mesoblastic nephroma is a benign tumor of spindle cells that may present infiltrative features (36). Rhabdoid tumor of the kidney and mesoblastic nephroma tumor, unlike RMC, occur during early childhood (average age of 11 and 3 months, respectively). Wilms’ tumor is the most common childhood renal cancer; this tumor is localized in the cortex, has an expansive growth, rounded shape and well-defined margins (33). Other renal tumors with infiltrative pattern are found in the elderly, for example, transitional cell carcinoma and sarcomatoid variants of RCC (33, 36). Infectious diseases such as acute bacterial nephritis may mimic the infiltrative pattern of RMC on imaging although the clinic-laboratory features are very different (21, 33). MRI, CT scan, and the anamnesis are important for the characterization of the lesions; CT is fundamental to the staging. However, histology must still be performed to confirm the diagnosis and to set up a proper treatment plan.

## Management of RMC

**Figure 1.**
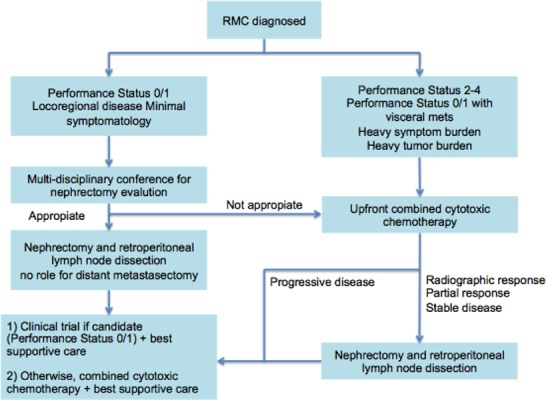
Treatment algorithm by Shah et al. (37). This algorithm suggests the need for a multidisciplinary approach for the effective management of renal medullary carcinoma.

A recent study (37) carried out on 52 patients described the management and outcomes of RMC patients, featuring predictors of overall survival. The vast majority of patients presented with advanced stage of disease, but three patients who had local disease, developed tumor recurrence within a few months after nephrectomy (median time of 3 months). In this study, the overall median survival was 13 months in comparison with the historic 5 months. The prognosis remained low, and <20% of patients survived for 24 months. However, in well-selected patients who respond to initial chemotherapy, or with low volume of metastases, nephrectomy can potentially play an important role also in the metastatic setting, and resection of all visible retroperitoneal disease should be the target of surgery. The data show that the nephrectomy may have a therapeutic benefit upfront or after response to chemotherapy. However, the decision to perform nephrectomy is influenced by many variables specific to the patient. A thorough patient assessment and a multidisciplinary deliberation are needed before a surgical treatment plan. In relation to these data, an algorithm has been recommended for RMC treatment (Figure 1) (37). The authors recommend the use of combined cytotoxic chemotherapy over single agents or targeted therapies, in particular for the palliation of symptoms. They also suggest the next frontier of RMC research is the molecular characterization in order to detect biologically relevant targets that can be exploited for the development of targeted therapies (37). As already mentioned, RMC is correlated with the loss of SMARCB1, a tumor suppressor gene on chromosome 22 (7, 38, 39). SMARCB1 acts as regulator of the remodeling of a repressor of transcription of cyclin D1. Therefore, the loss of SMARCB1 leads to an increase of the transcription of cyclin D1 in RMC (40). This results in the overexpression of Enhancer of zeste homolog 2 (EZH2) (a histone methyltransferase). For this reason, EZH2 inhibitors such as tazemetostat are used as therapeutic options (7, 41).

## Conclusion

RMC is a rare, highly aggressive tumor that occurs mainly in African-American adolescent and young adult patients with SC hemoglobinopathies. A summary of imaging studies published between 1995 and 2016 shows that the highest number of cases of RMC were evaluated through CT. The most frequent CT features were intratumoral necrosis, right renal location, lymphadenopathy, caliectasis, and poorly defined margins of the tumor. The features such as areas of necrosis and lymph node involvement correlate with the typical histological features of RMC. Few cases have been evaluated through ultrasound and MRI, and no case through CEUS. Further studies, including different imaging techniques, are warranted to define the characteristics of this aggressive tumor.

## Conflicts of interest

The authors declare no potential conflicts of interest with respect to research, authorship and/or publication of this article.
